# Structure and mechanical properties of *Octopus vulgaris* suckers

**DOI:** 10.1098/rsif.2013.0816

**Published:** 2014-02-06

**Authors:** Francesca Tramacere, Alexander Kovalev, Thomas Kleinteich, Stanislav N. Gorb, Barbara Mazzolai

**Affiliations:** 1 Center for Micro-BioRobotics@SSSA, Istituto Italiano di Tecnologia, Pontedera, Italy; 2 Functional Morphology and Biomechanics, Zoological Institute, Kiel University, Kiel, Germany

**Keywords:** octopus suckers, suction cup, elasticity modulus, viscoelasticity, bioinspiration

## Abstract

In this study, we investigate the morphology and mechanical features of *Octopus vulgaris* suckers, which may serve as a model for the creation of a new generation of attachment devices. Octopus suckers attach to a wide range of substrates in wet conditions, including rough surfaces. This amazing feature is made possible by the sucker's tissues, which are pliable to the substrate profile. Previous studies have described a peculiar internal structure that plays a fundamental role in the attachment and detachment processes of the sucker. In this work, we present a mechanical characterization of the tissues involved in the attachment process, which was performed using microindentation tests. We evaluated the elasticity modulus and viscoelastic parameters of the natural tissues (*E* ∼ 10 kPa) and measured the mechanical properties of some artificial materials that have previously been used in soft robotics. Such a comparison of biological prototypes and artificial material that mimics octopus-sucker tissue is crucial for the design of innovative artificial suction cups for use in wet environments. We conclude that the properties of the common elastomers that are generally used in soft robotics are quite dissimilar to the properties of biological suckers.

## 1. Introduction

The octopus is an emblem of soft robotics because it consists of no rigid structure with redundant degrees of freedom. The octopus arms show the ability to bend in all directions, to produce very fast elongations, and to change its stiffness. Various groups have investigated and mimicked the exceptional flexibility of octopus arms. The artificial requirements of soft robotic arms have been discussed for designing innovative technological solutions [1] and different possible actuation principles have been analysed [2]. Up to the present, some artificial manipulators have been designed and developed: continuum robot arms based on electroactive polymers and pneumatic systems [3], robotic tentacles with three-dimensional mobility made of flexible elastomers [4], cable driven mock-ups [5] and soft robot arms actuated with shape memory alloy springs [6]. In addition, a multi-purpose platform has been developed in order to test bioinspired mock-ups [7]. In recent years, octopus suckers have become the focus of investigations because of their substantial capability to generate large attachment forces on non-porous surfaces [8–11] and because of their ability to maintain these forces for rather long periods of time without any muscular energy consumption [12–14]. The morphology and physiology of octopus suckers have been previously investigated [11,13,14], their attachment forces have been measured or estimated [15,16] and the coordination between different suckers has been analysed [17]. Some bioinspired artificial devices have already been developed [9] on the basis of these studies, but the capabilities of these devices are still far from being comparable to the attachment performance of real octopus suckers.

Despite these extensive efforts, no studies have been conducted concerning the mechanical properties of octopus sucker materials, which play an important role in generating efficient attachment because of the softness of the tissues, and therefore their compliance to the substrate. The infundibulum can closely match the contour of a surface, and thereby provide a watertight seal. In this study, we analysed the general morphology of the sucker and measured the mechanical properties of its tissues. This information is a fundamental necessity for the extraction of the requirements for designing and developing an artificial suction cup that can better mimic its natural prototype.

## 2. Material and methods

### 2.1. Experimental animals

Adult specimens of *Octopus vulgaris* were obtained, already dead, from licensed fishermen who usually capture them for human consumption. The animals that were used for the morphological investigations were captured from the wild in the bay of Livorno in October 2012. The animals that were used for the biomechanical investigations were captured from the wild in the Tyrrhenian Sea in November 2012. In all cases, the suckers were explanted when the animals were already dead.

### 2.2. Morphology of octopus suckers

For microcomputed tomography (micro-CT), freshly explanted suckers were fixed in 95% ethanol. Before the experiment, the specimens were hydrated in decreasing ethanol concentrations of 95% followed by 70, 50 and 30%, and then in distilled water for 4 h each. After rehydration, the specimens were treated overnight in a contrast solution (1% Lugol's iodine–potassium–iodide) and rinsed in distilled water for 4 h. For scanning, each individual specimen was placed in a Plexiglas jar filled with distilled water. We fixed the specimen to the jar using needles to prevent any movement so that the sample remained suspended in the jar. The experiments were performed using a SkyScan 1172 HR micro-CT (Bruker microCT, Kontich, Belgium). The sucker was scanned throughout its volume. After the first scan, we extracted some portions of the sucker and higher resolution scans of these portions were performed.

For light microscopy, freshly explanted suckers were fixed in 95% ethanol. Samples of few millimetres were extracted from the acetabular and infundibular portions and critical-point dried using a critical-point-drying apparatus (E3000 Series, Quorum Technologies, UK). The dried samples were mounted on aluminium stubs, sputter coated with 10 nm of gold–palladium (SCD 500 Sputter Coater equipped with a QSG 100 Quartz Film Thickness Monitor, BAL-TEC, Liechtenstein) and viewed using a Zygo New View 6000 (Zygo Corporation, Middlefield, CT, USA) white-light interferometer.

### 2.3. Mechanical properties of octopus suckers and artificial materials

**Figure 1.**
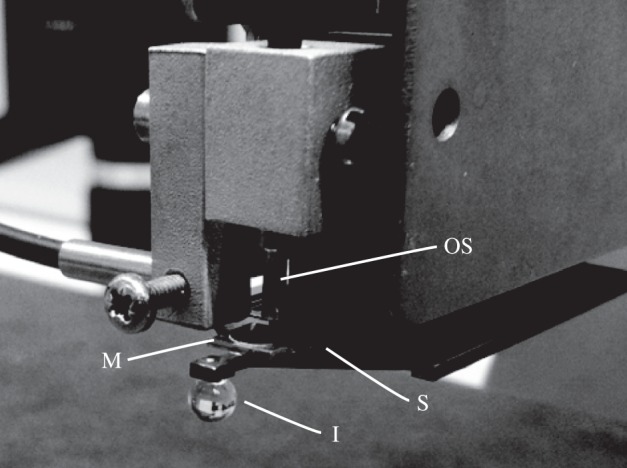
Microindentation unit. The micro-force tester used to measure the mechanical properties of natural suckers and artificial materials. The spherical micro-indenter (I) is attached to a spring (S), driven by a motor and pressed against the soft sample. The deflection of the spring is monitored by the fibre-optic sensor (OS) using monochromatic light sent to and reflected from the mirror (M). The collected data were transmitted to a computer with a sampling frequency of 10 Hz.

The mechanical properties of the infundibular and acetabular portions of 10 *O. vulgaris* suckers and of three soft elastomeric materials that have been used for the fabrication of soft robotic components (Ecoflex 00–10, Ecoflex 00–30 and Dragon Skin 10 (all from Smooth On, USA)) [5,6] were measured using a Basalt-BT01 micro-force tester (Tetra GmbH, Ilmenau, Germany) [18]. This instrument consists of three main components: a platform, a spring and a fibre-optic sensor. The platform holds the clamped sample, and a motorized stage moves the platform vertically at speeds ranging from 15 to 80 µm s^−1^. For the mechanical tests, the spring was equipped with a 1.5 mm diameter spherical indenter (as shown in figure 1). Two different springs were used for the investigation of the natural and artificial samples. The spring constants were 73 and 256 N m^−1^ for the natural and artificial samples, respectively. The entire set-up was placed on a massive granite table, and all measurements were performed at room temperature (18–21°C) and a relative humidity of 65–78%. To prevent the desiccation of the natural specimens, we stored the octopus arms with the suckers attached in seawater until the measurement was finished.

**Figure 2.**
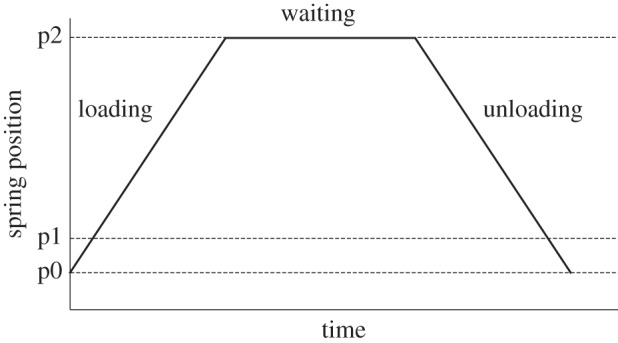
Spring position as a function of time in a typical indentation test. The solid line indicates the position of the spring with the attached spherical indenter during the measurements; p0 is the starting point of the spring; p1 indicates the level of the spring at the sample surface, which is the starting point of indentation into the sample; and p2 is the position of the spring at the maximum indentation depth reached by the indenter. In accordance with what was said, (p2-p0) and (p2-p1) are spring displacement and specimen indentation, respectively.

For each measurement, the indenter was brought to an upward position of approximately 100 µm from the sample surface. The indentation procedure consisted of three phases (figure 2). In the loading phase, the spring was moved downwards at constant velocity and the indenter was pressed against the sample surface. In the waiting phase, the indenter and the sample surface were held in contact under a specified load for 10 s. In the unloading phase, the indentation unit was removed from the sample surface at constant velocity. After each measurement, the indenter was cleaned with acetone and rinsed with distilled water. The spring deflection was monitored using a fibre-optic sensor; force, time and displacement data were continuously acquired with a sample frequency of 10 Hz.

Each sample, both natural and artificial, was investigated using three different spring displacements (200 µm apart) and five different velocities of indentation (15, 20, 40, 60 and 80 µm s^−1^) with a recovery time of 3 min between two consecutive measurements. In the case of natural samples, during the recovery time, the specimens were continuously hydrated with seawater. Because the spring deforms with the sample during measurement, the deflection of the spring from the control data (when the spring was pressed on a hard sample) was subtracted to obtain the actual force–displacement curve.

The effective elasticity modulus (*E*_s_) (analogous to the elasticity modulus of a homogeneous material) [19] was calculated using the Hertz model [20]. The formula used for fitting the force–deformation (*F*(*t*)−**δ**(*t*)) curves was as follows:2.1
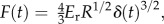
with2.2
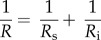
2.3
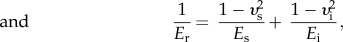
where *E*_r_ is called the reduced modulus; *E*_s_, *v*_s_, and *R*_s_ are the elastic modulus, Poisson ratio and radius of the sample, respectively; and *E*_i_, *v*_i_ and *R*_i_ are the same parameters for the indenter.

For the calculations, because the material of the indenter (sapphire sphere) was considerably stiffer than the materials of the samples (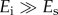) and the radii of curvature of the sample surfaces were considerably greater than the radius of curvature of the indenter's tip (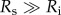), *R*_s_ can be considered to be an infinite radius2.4
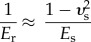
2.5
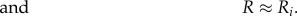


Because we do not know the Poisson's ratio of octopus tissues, we assumed that the octopus tissues were incompressible (Poisson's ratio = 0.5), in accord with the typical behaviour of elastomers, to calculate the elasticity modulus.

**Figure 3.**
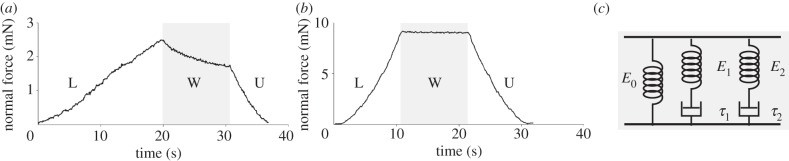
Force–time curves. Typical force–time curves for the (*a*) biological and (*b*) artificial samples obtained using the micro-force tester. During the loading phase (L), the indenter was pressed against the sample, thus increasing the compression force. Then, during the waiting phase (W), the indenter was maintained in contact with the sample for a fixed amount of time. During this period of time, a rapid decrease in the force was detected only in the natural samples. This result highlights the viscoelastic behaviour of the natural tissues. During the unloading phase (U), the indenter was driven away from the sample. (*c*) The dashpot–spring model used to fit the force–time curves during the waiting phase for the natural samples.

During the waiting phase, when the indenter was kept in contact with the sample without any movement, an exponential decrease in the force was observed only in the measurements of the natural specimens (figure 3*a,b*). This decrease in the force is a typical behaviour of viscoelastic materials. The relaxation profile in the force–time curve was fitted using a linear solid model [21,22], in which the initial time point corresponds to the maximal load before relaxation. The model includes two sets of serial dashpots and springs (**τ**_1_, *E*_1_, **τ**_2_ and *E*_2_) and an additional spring (*E*_0_) in parallel with the two dashpot–spring sets (figure 3*c*). The dashpots reflect the relaxation profile, whereas the three springs correspond to the elasticity modulus of the entire system. Assuming that the strain (**ɛ**) is essentially constant in the waiting phase, the stress (**σ**) can be calculated as follows:2.6
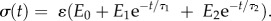

The contact area (*A*) derived from the Hertz theory was calculated as follows:2.7
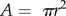
2.8
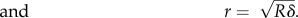


Thus, the formula for the force–time dependence (*F*(*t*)) was2.9
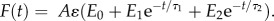


Because we conducted measurements using various spring displacements and indentation velocities, we normalized the force data to the first force value (*F*(*t*_0_)) recorded during the waiting phase to allow the comparison of homogeneous datasets.

The formula used for fitting the normalized force–time (*F_n_*(*t*)) curves was2.10
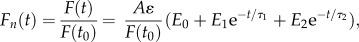
2.11
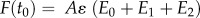
2.12
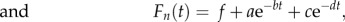
where2.13
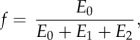
2.14
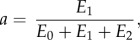
2.15
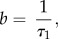
2.16
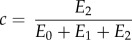
2.17
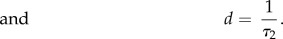
The parameter *f* represents the normalized force at *t* → ∞; *b* and *d* are the inverses of the time constants **τ**_1_ and **τ**_2_, respectively; and the sum of *a*, *c* and *f* corresponds to the value of the normalized force at the initial moment of time (*t* = 0).

**Figure 4.**
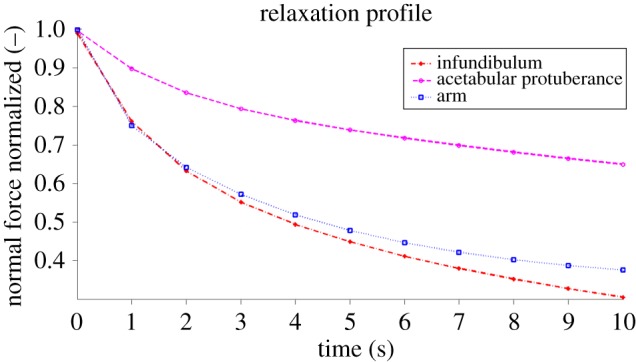
Relaxation curves obtained for different octopus tissues. The curves demonstrate the viscoelastic behaviour of the infundibulum, the acetabular protuberance and the dorsal tissue of the arm during the relaxation phase. The data were obtained by deforming each sample with a spherical indenter up to a predefined normal force (4.5 mN) at a constant indentation velocity (60 µm s^−1^). This procedure was repeated three times for each octopus tissue (infundibulum, acetabulum and dorsal portion of the arm). The curves in the figure represent the standard linear solid viscoelastic models used to fit the force–time data. It is apparent that the infundibular behaviour is quite similar to the behaviour of the dorsal arm tissue; both differ from the relaxation profile of the acetabular protuberance, which decreases more slowly than the other two. Considering the plots above, for an applied normal force of 4.5 mN, there was a 35, 63 or 70% relaxation of the force after 10 s for the acetabular protuberance, the infundibulum or the arm, respectively. Based on these results, it can be concluded that for this initial applied normal force, the acetabular protuberance behaves more elastically and less viscously than the infundibulum and the dorsal tissue of the arm. The responses of these last two tissues exhibit similar behaviour.

To study the differences in the viscoelastic properties of the sucker tissues with respect to another octopus tissue (the dorsal portion of the arm was chosen), we designed an experiment in which each sample was deformed by the spherical indenter up to a predefined normal force with a constant indentation velocity. This procedure was repeated three times for each octopus tissue (the infundibulum, the acetabulum and the dorsal portion of the arm). The results are shown as force–time curves in figure 4.

## 3. Results

### 3.1. Morphology of octopus suckers

**Figure 5.**
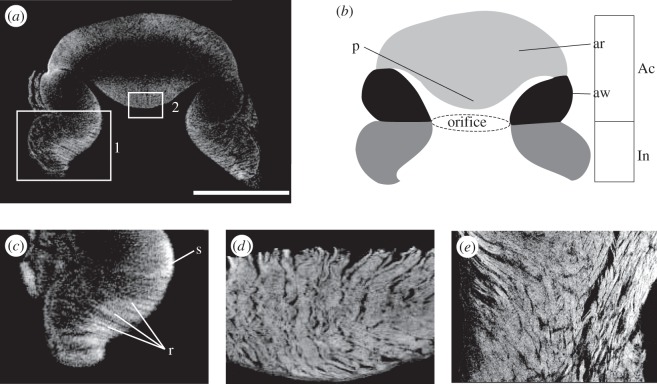
Octopus-sucker structure. (*a*) Microcomputed tomography model of the sucker virtually dissected in the transverse plane. The scale bar corresponds to 1 cm. (*b*) A schematic of (*a*) with the main structures of the sucker highlighted, including the acetabulum (Ac), which is the hollow upper portion and consists of the acetabular wall (aw) and the acetabular roof (ar). The acetabular roof is characterized by an evident protuberance (p). Meanwhile, the lower portion, which comes into contact with the substrate during contact formation and attachment, is the infundibulum (In). The transition between these two structures is the so-called orifice. (*c*) Detail of white box 1 in (*a*) (x2). We can recognize the radial muscular bundles (r) of the infundibular portion and the primary sphincter (s), which corresponds to the orifice. (*d,e*) Transverse (*d*) and oblique (*e*) plane of white box 2 in (*a*) (x6). In (*d*), we can recognize the radial muscular bundles of the acetabular protuberance. In (*e*), we can recognize the following: on the left side, the radial muscular bundles and on the right side, the cross-connective tissue fibres that intersect with the radial muscles.

*Octopus vulgaris* presents two rows of suckers along its arms. The diameters of the suckers range from few centimetres (in the proximal tract) to millimetres (in the distal tract). The octopus sucker is a muscular-hydrostat [23] that consists of two portions: an upper hollow structure, the acetabulum, which in turn consists of a domed roof (in the upper part) and a wall region (in the remaining parts), and a disc-like portion, the infundibulum (figure 5*a,b*). These two portions are connected by an orifice. The sucker is completely encircled by a connective-tissue layer, and the infundibulum presents a surrounding rim of epithelium. As was found in [14], the acetabulum of *O. vulgaris* presents an evident protuberance on its roof that fills approximately 80% of its internal volume (figure 5), which differs from other octopus species, in which the acetabulum appears as a hollow spherical cup without any protuberance [12,13]. The infundibulum and acetabular wall are characterized by a three-dimensional array of muscles: radial, circular and meridional fibres. The radial fibres, which cross the entire thickness of the sucker, are uniformly distributed throughout the structure (figure 5*a,c*); the circular fibres are located in the inner part (closer to the external surface) of the infundibulum and in the outer part of the acetabular wall (farther from the external surface), and the meridional fibres are located in the central-outer part of the infundibulum and in the outer part of the acetabular wall [12–14]. The acetabular roof lacks circular fibres; it consists mainly of radial muscular fibres (figure 5*d*), with some meridional fibres confined to the apex (top part of the acetabulum) [12–14]. However, the acetabular roof is the only portion of the entire sucker that presents a dense network of cross-connective fibres intersecting with the muscular radial fibres (figure 5*e*). These observations confirm what was found in octopus suckers in previous studies [12–14]. Nevertheless, in this work as reported below, we provide for the first time a quantitative information of the surface features of the octopus suckers.

**Figure 6.**
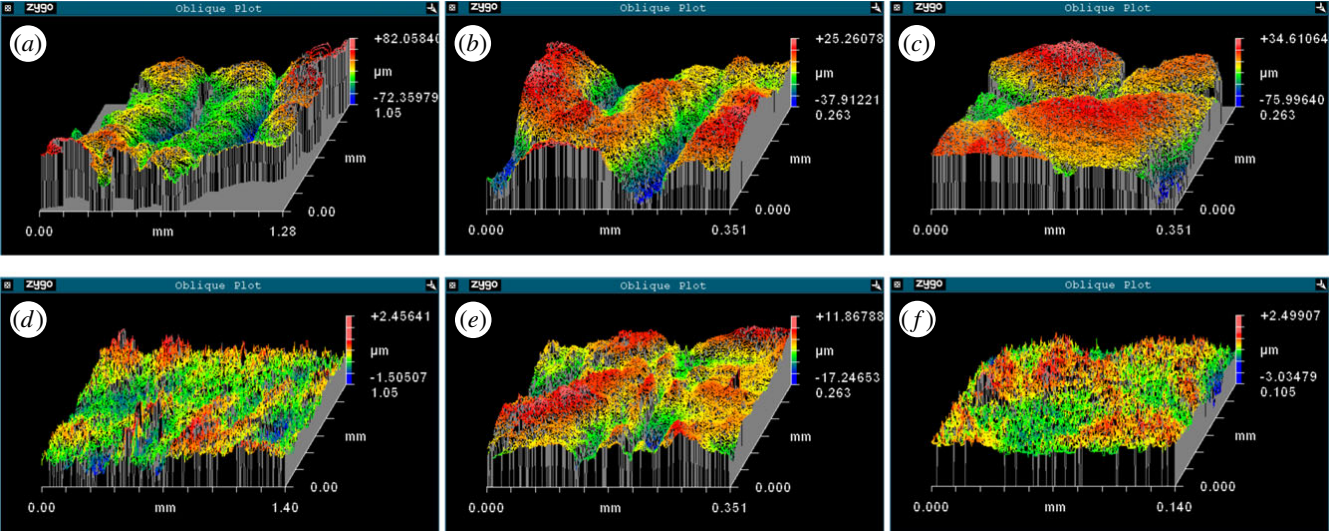
Three-dimensional white-light interferometric images of the surfaces of the infundibular (*a–c*) and the acetabular protuberance (*d–f*) of dry samples. (*a*) Circumferential grooves. (*b*) Radial grooves. (*c*) An intersection between radial and circumferential grooves. (*d–f*) Different magnifications of the roughness of the acetabular protuberance. For quantitative data, please refer to table 1.

The surface of the infundibular portion presents circumferential and radial grooves and is covered with a chitinous cuticle that is continuously renewed. The circumferential grooves are concentrically spaced 280 ± 50 µm (mean ± s.d.) apart (figure 6*a*). By contrast, the radial grooves run from the orifice to the rim with an angular distribution of 18° ± 3° (considering 25 suckers with a diameter of 1.3 ± 0.7 cm); some of them branch again before reaching the rim (figure 6*b*). Circumferential grooves interdigitate radial ones, as shown in figure 6*c*.

The cuticle, which comes into contact with the substrate during attachment, bears numerous denticles. On average, the cuticle has a mean roughness (*R*_a_) of 11.3 ± 3.0 µm and a maximum height (*R*_z_) of 89.5 ± 20.0 µm (*N* = 31). The maximum height (*R*_z_) can be assumed to be the measure of the depth of the infundibular grooves.

The surface of the acetabular protuberance is completely different and exhibits a mean roughness (*R*_a_) of 1.3 ± 1.0 µm and a maximum height (*R*_z_) of 14.5 ± 10.6 µm (*N* = 25).

Table 1 gathers quantitative data of the samples shown in figure 6 (some examples of infundibular (figure 6*a–c*) and acetabular protuberance's surface (figure 6*d–f*), respectively).

**Table 1.** Quantitative data of samples shown in figure 6. M, magnification; *R*_z_, maximum height or peak-valley value; *R*_a_, mean roughness; *S_x_*, width of the investigated area; *S_y_*, height of the investigated area.

	M	*R_z_* (µm)	*R*_a_ (µm)	*S_x_* (mm)	*S_y_* (mm)
figure 6*a*	5×	154.4	19.2	1.28	1.05
figure 6*b*	20×	63.2	10.3	0.35	0.26
figure 6*c*	20×	110.6	11.1	0.35	0.26
figure 6*d*	5×	4.0	0.3	1.40	1.05
figure 6*e*	20×	29.1	2.8	0.35	0.26
figure 6*f*	50×	5.5	0.4	0.14	0.11

### 3.2. Mechanical properties of sucker tissues

In the measurements of the natural suckers, the average indentation during the loading phase was 370.4 ± 46.4 µm (mean ± s.d.) in the infundibular portion and 399.1 ± 63.7 µm in the acetabular protuberance, corresponding to compression forces of 3.4 ± 1.3 and 7.4 ± 2.1 mN, respectively. Table 2 summarizes the measured elasticity moduli for the infundibular and acetabular portions.

**Table 2.** Elasticity moduli. Elasticity moduli (mean ± s.d., kPa) of the infundibular and acetabular-protuberance portions of *O. vulgaris* suckers (*N* = 10). Measurements were performed using five different indentation velocities and three different spring displacements (with **δ** = 200 µm). Each value of spring displacement (*Δ*0, *Δ*0 + **δ**, *Δ*0 + 2**δ**) corresponds to the quantity p2-p0 in figure 2.

	indentation velocity (µm s^−1^)				
spring displacement (µm)	15	20	40	60	80
infundibulum					
*Δ*_0_	6.0 ± 1.7	6.8 ± 1.9	7.1 ± 1.9	7.2 ± 1.9	7.3 ± 1.7
*Δ*_0_ + **δ**	6.5 ± 1.8	7.0 ± 1.8	8.3 ± 2.3	9.1 ± 2.6	9.6 ± 2.9
*Δ*_0_ + 2**δ**	6.9 ± 2.5	7.4 ± 2.6	8.0 ± 2.9	8.6 ± 2.7	9.3 ± 3.0
acetabular protuberance					
*Δ*_0_	15.5 ± 6.3	16.6 ± 6.5	18.3 ± 6.4	19.9 ± 6.7	20.8 ± 6.9
*Δ*_0_ + **δ**	16.0 ± 4.0	16.7 ± 4.4	18.7 ± 5.8	20.3 ± 6.4	21.5 ± 7.1
*Δ*_0_ + 2**δ**	14.6 ± 5.0	15.1 ± 5.5	16.4 ± 6.2	18.7 ± 7.0	23.2 ± 8.9

Two-way analysis of variance (ANOVA) was performed considering the different spring displacements and indentation velocities and revealed that the elasticity modulus varied significantly with the spring displacement and indentation velocity. This behaviour was observed for both the infundibular and acetabular-protuberance tissues (*p* < 0.05), with the exception that the elasticity modulus of the acetabular protuberance exhibited no sensitivity to different spring displacements (*p* = 0.28) (table 3). The elasticity modulus (*E*) was always greater in the acetabular protuberance than in the infundibulum, indicating that the acetabular protuberance is stiffer than the infundibulum. To compare these values to those of another octopus tissue, we performed the same measurement on the dorsal portion of an octopus arm. We found that the elasticity modulus of the arm was 13.2 ± 3.7 kPa. The measured values revealed that the dorsal tissue of the octopus arm is stiffer than the infundibulum but softer than the acetabular protuberance. The data from this dorsal part of the arm showed significant variability in the value of *E* with the spring displacement ((*E*) *F* = 8.33, *p* = 0.01) but not with the indentation velocity ((*E*) *F* = 0.25, *p* = 0.9).

**Table 3.** Two-way ANOVA of the data concerning the properties of octopus-sucker tissues. Statistical analysis of possible differences in the elasticity modulus associated with the use of different spring displacements and indentation velocities in the infundibulum and the acetabular protuberance (*F* parameter refers to *F*-test of Fisher).

	infundibulum		acetabular protuberance	
	displacement	velocity	displacement	velocity
elasticity modulus	*F* = 11.71, *p* < 0.01	*F* = 12.41, *p* < 0.01	*F* = 1.49, *p* = 0.28	*F* = 22.82, *p* < 0.01

Table 4 summarizes the mean values of the five parameters of the standard linear solid viscoelastic model used to fit the data from the infundibulum and acetabular protuberance. To determine the differences in the viscoelastic properties associated with different spring displacements and indentation velocities, a two-way ANOVA was performed for the five parameters of the model (table 5). The analysis demonstrated that in the case of the infundibular tissue, all five parameters varied significantly (*p* < 0.05) with the spring displacement but not with the indentation velocity; in the case of the acetabular-protuberance tissue, all five parameters varied significantly (*p* < 0.05) with both the spring displacement and the indentation velocity, with the exception of the b parameter (the inverse of the time constant **τ**_1_), which was not sensitive to different indentation velocities (*p* = 0.40).

**Table 4.** Viscoelastic parameters of octopus-sucker tissues. Mean values ± s.d., considering different indentation velocities and different spring displacements, of the parameters of the standard linear solid viscoelastic model applied to measurements of the infundibulum and acetabular protuberance of octopus suckers. For the definitions of parameters *a*, *b*, *c*, *d* and *f*, please refer to equations (2.12)–(2.17).

	infundibulum	acetabular protuberance
*a*	0.30 ± 0.24	0.16 ± 0.05
*b*	0.83 ± 0.31	0.66 ± 0.10
*c*	0.57 ± 0.10	0.52 ± 0.03
*d*	0.12 ± 0.14	0.04 ± 0.01
*f*	0.13 ± 0.36	0.31 ± 0.02

**Table 5.** Two-way ANOVA of viscoelastic parameters of octopus-sucker tissues. Analysis of possible differences in the parameters of the standard linear solid viscoelastic model associated with the use of different spring displacements and indentation velocities (*F* parameter refers to *F*-test of Fisher). For the definitions of parameters *a*, *b*, *c*, *d* and *f*, please refer to equations (2.12)–(2.17).

	infundibulum		acetabular protuberance	
	displacement	velocity	displacement	velocity
*a*	*F* = 6.63, *p* = 0.02	*F* = 0.39, *p* = 0.81	*F* = 105.10, *p* = 0	*F* = 34.65, *p* = 0
*b*	*F* = 6.31, *p* = 0.02	*F* = 0.53, *p* = 0.71	*F* = 6.75, *p* = 0.02	*F* = 1.14, *p* = 0.40
*c*	*F* = 5.34, *p* = 0.03	*F* = 2.33, *p* = 0.14	*F* = 56.42, *p* = 0	*F* = 11.82, *p* = 0
*d*	*F* = 6.63, *p* = 0.02	*F* = 0.8, *p* = 0.56	*F* = 72.80, *p* = 0	*F* = 5.5, *p* = 0.02
*f*	*F* = 6.05, *p* = 0.03	*F* = 0.79, *p* = 0.56	*F* = 86.80, *p* = 0	*F* = 19.07, *p* = 0

Figure 4 shows the differences in the viscoelastic profile during the waiting phase of the two tissues tested (the infundibulum and acetabular protuberance) compared with the profile found for the dorsal arm tissue. We observed that the profile of the infundibulum is more similar to the profile of the dorsal arm tissue than to the profile of the acetabular protuberance.

### 3.3. Mechanical properties of artificial elastomeric materials

During the measurements of the artificial materials, a gradual indentation occurred during the loading phase. On average, the indentation was 242.7 ± 38.0 µm in Ecoflex 00–10, 214.7 ± 87.4 µm in Ecoflex 00–30 and 174.3 ± 145.3 µm in Dragon Skin 10, corresponding to compression forces of 13.1 ± 0.5, 29.1 ± 0.2, and 80.9 ± 0.9 mN, respectively. Table 6 summarizes the measured elasticity moduli for the three artificial materials. The data obtained from the measurements of the artificial materials exhibited significant variability in the elasticity modulus (*E*) with the spring displacement (*p* < 0.01) but not with the indentation velocity, except for the Dragon Skin 10 material, which exhibited significant variability with the indentation velocity (*p* = 0.02) (table 7).

**Table 6.** Evaluation of artificial materials. Elastic moduli (mean ± s.d., kPa), considering different indentation velocities and different spring displacements, of three different artificial rubber-like materials tested for comparison with the octopus tissues.

	artificial material		
	Ecoflex 00–10	Ecoflex 00–30	Dragon Skin 10
elastic modulus	16.3 ± 2.1	34.8 ± 2.7	129.0 ± 9.7

**Table 7.** Two-way ANOVA of properties of artificial materials. Analysis of possible differences in the elasticity modulus associated with the use of different spring displacements and indentation velocities in three artificial materials (*F* parameter refers to *F*-test of Fisher).

	Ecoflex 00–10		Ecoflex 00–30		Dragon Skin 10	
	displacement	velocity	displacement	velocity	displacement	velocity
*E*	*F* = 39.72, *p* < 0.01	*F* = 2.95, *p* = 0.09	*F* = 24.93, *p* < 0.01	*F* = 1.03, *p* = 0.45	*F* = 14.79, *p* < 0.01	*F* = 5.90, *p* = 0.02

Because we did not define the Poisson's ratio of these artificial materials, we assumed that the artificial materials were incompressible (Poisson's ratio = 0.5), in accord with the typical behaviour of elastomers, to calculate the elasticity modulus.

No viscoelastic behaviour was observed during the waiting phase for the artificial materials (figure 3*b*).

## 4. Discussion

### 4.1. Morphology and physiology of octopus suckers

The infundibulum portion is characterized by radial grooves with a mean angular distribution of 18° and by concentric grooves with a mean spacing of 280 µm. These grooves present a mean depth of 89.5 µm. This morphology has already been observed in previous works [12–14] but it was not previously quantified. The structure of the infundibular surface is fundamental to its ability to increase its contact area with the substrate during attachment. This network of grooves allows low pressure, which is generated in the acetabular chamber, to be transmitted to almost the entire sucker–substrate interface. This structured surface is completely different from that of the common artificial suction cup, in which the surface is completely smooth, and thus could be taken into account as a bioinspiration model for the design of prototypes that are intended to operate under the same conditions as octopus suckers.

This study supports the characteristic morphology of the acetabular roof in *O. vulgaris* that was previously found in [14]. We have demonstrated the presence of a protuberance that protrudes towards the sucker's orifice (figure 5) and measured the roughnesses of the acetabular part (*R*_a_ = 1.3 µm) and the infundibulum (*R*_a_ = 11.3 µm). The measurement of the infundibular roughness is also useful in the design of suction-cup prototypes for use in wet conditions. Mimicry of the infundibular morphology by an artificial suction cup should guarantee the maximum attachment area and good resistance to shear forces. The roughness found on the acetabular protuberance surface confirms the morphology that was previously observed in [14], in contrast with earlier works, in which the acetabulum was described as having a completely smooth surface [12,13]. This finding, combined with the knowledge of the specific infundibular roughness, is particularly important because it represents, to the best of our knowledge, the first quantitative data concerning the roughness of octopus-sucker tissues. Moreover, our measurements provide further details to improve the attachment hypothesis suggested in [14]. In accordance with this hypothesis, the acetabular roughness could play a role in achieving the watertight closure of the orifice to allow energy-saving attachment for long periods of time.

### 4.2. Mechanical properties of natural suckers and artificial materials

The measurements of the mechanical properties of octopus suckers demonstrated that the tissues are composed of a very soft material: we measured mean elasticity moduli of 7.7 and 18.1 kPa for the infundibulum and the acetabular protuberance, respectively. Octopus-sucker tissues are thus among the softest biological materials, equivalent to coelenterate mesoglea or jellyfish jelly (*E* ∼ 10 kPa [24]). Although sucker tissues are less soft than brain tissue (*E* ∼ 1 kPa [25]) and adipose tissue (*E* ∼ 3 kPa [26]), the elasticity values found in this study are much lower than those of other soft biological materials, such as human skin (*E* ∼ 150 kPa [27]), aorta tissue (*E* ∼ 500 kPa [28]), the aortic valve (*E* ∼ 1 MPa [29]) and the chordae tendineae of the heart valve (*E* ∼ 50 MPa [30]). Moreover, the elastic moduli of octopus-sucker tissues are comparable to those obtained for tree frogs (*E* ∼ 4–25 kPa [31]), and they are slightly lower than those of grasshoppers (*E* ∼ 20–65 kPa [32,33]) and caeliferan insects (*E* ∼ 250–750 kPa [34]). Particularly interesting is the similarity between the elasticity modulus of the infundibulum and that of echinoderm tube feet (*E* ∼ 6–8 kPa [35]), as both systems work under similar wet conditions and are able to attach to rough substrates.

Our experiments showed that the elasticity modulus was higher for the acetabular protuberance than for the infundibulum. The higher stiffness of the acetabular protuberance could be a consequence of the presence of cross-connective tissues, which are present in the acetabular protuberance but absent in the rest of the sucker's tissues. In the acetabular protuberance, a dense network of cross-connective tissues is intersected with the muscular fibres, while the infundibulum consists entirely of muscles.

A significant difference (*p* < 0.05) in the elasticity modulus of the infundibulum was also observed for different spring displacements. The experiments showed an increasing elasticity modulus, as the spring displacement increased. The infundibular tissue was particularly soft at low displacements and became stiffer at higher displacements. To explain this result, we must consider that the outer layer of the infundibulum consists of epithelium (approx. 40 µm) and that just below, before the muscular tissue, there is a layer of connective tissue (approx. 15 µm). Thus, the stiffness measured at low displacements could be related to the epithelium and the connective-tissue layer. The soft properties of these outer layers are critical, considering that the infundibulum is the only portion of the suckers that must be absolutely compliant when it comes into contact with substrates of various roughnesses to achieve a perfect seal. The elasticity modulus measured at higher displacements is likely related to the underlying muscular tissue. The acetabular protuberance, which did not exhibit a significantly different response to various spring displacements (*p* = 0.28), presents a thinner epithelium (approx. 10 µm) and layer of connective tissue (approx. 10 µm). These layers are likely not thick enough to be probed by the experimental set-up. Therefore, the elasticity modulus measured for the acetabular protuberance is likely related only to the dense network of muscular and cross-connective fibres. Significant differences (*p* < 0.05) in the elasticity moduli of both the infundibulum and acetabular protuberance were observed for different indentation velocities. Both sucker tissues behaved as soft materials at low indentation velocities and became stiffer at high indentation velocities. This behaviour of the sucker tissues could be related to the attachment function of the system. They must be particularly soft during contact formation to allow good pliancy against the substrate. Afterwards, during the suction process, the indentation velocity of the sucker tissues against the substrates strongly increases because of the low pressure under the suction cup. To maintain attachment and to resist external stresses, the sucker tissues must become stiffer in this phase than during the initial contact with the substrate.

The measured properties of the artificial materials were considerably different from those of the sucker materials, except for the fact that the elastic modulus of the Dragon Skin 10 was also sensitive to different indentation velocities. Contrary to the artificial elastomeric materials, which exhibit elastic behaviour, the octopus-sucker tissues are characterized by viscoelastic behaviour. Viscoelasticity has also been observed in many other natural fibrous composites [31,33,35–39].

The viscoelastic properties of the octopus tissues were confirmed by the analysis of the relaxation curves. The relaxation profile during the waiting phase was fitted using a standard linear solid viscoelastic model with five parameters (equation (2.12)). The model used for the calculation of the viscous behaviour was the same as the one previously used for grasshoppers [33]. This viscoelasticity model takes into account the fact that relaxation does not occur at a single unique time but at a combination of several times. The octopus-sucker tissues were characterized by two different relaxation time constants (RTC: **τ**_1_ and **τ**_2_), one long, more elastic RTC (**τ**_2_) and one short, more viscous RTC (**τ**_1_). The infundibulum exhibited a rapid relaxation (**τ**_1_ = 1.20 s) that can likely be attributed to the soft tissues of the outer layers and a slow relaxation (**τ**_2_ = 8.33 s) that can likely be attributed to the underlying muscular arrangement.

A two-way ANOVA demonstrated that all five parameters of the infundibular tissue were sensitive (*p* < 0.05) to the spring displacement but insensitive (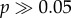) to the indentation velocity. Considering the elaborated arrangement of the sucker tissues, as the spring displacement varies, different combinations of the underlying muscular fibres become involved in the relaxation phase. Moreover, for each displacement, a certain initial value of applied force (equal to the sum of *a*, *c* and *f* parameters) is induced, and a specific relaxation response is measured. The same structure exhibits nearly the same general relaxation response for various indentation velocities. By contrast, all five parameters of the acetabular protuberance were sensitive to both the spring displacement and the indentation velocity, except parameter *b* (the inverse of the time constant **τ**_1_), which was insensitive to different indentation velocities. The acetabular protuberance exhibited a rapid relaxation (**τ**_1_ = 1.52 s) that can likely be attributed to the soft tissues of the outer layers of the acetabular protuberance. This structure is very similar to the outer layers of the infundibulum, aside from the thickness, and behaves similarly in terms of its RTC and insensitivity to the indentation velocity. The slow relaxation of the acetabular protuberance (**τ**_2_ = 25 s), meanwhile, can likely be attributed to the underlying dense network of muscles and connective fibres.

The primary difference in the behaviour of the two sucker portions was observed for the slow RTC (**τ**_2_), which is likely related to the underlying tissues, which are characterized by different compositions. While the infundibulum is supported by a three-dimensional array of muscles, the acetabular protuberance mainly contains only one type of muscles (radial muscles), in which cross-connective tissue fibres are densely embedded. Considering the observed results, the structure of the muscular and connective tissue fibres appears to be more elastic than the structure that consists only of muscular fibres. Although the role of the cross-connective tissue is completely unknown, the results reported here fit well with the hypothesis that these fibres, which are embedded in muscular tissue, are able to store elastic energy to generate attachment for a long period of time without muscle contractions [12,13].

Based on our results, the more viscous behaviour (**τ**_1_) should be typical of the outer layers of the infundibulum and the acetabular protuberance. Both these two portions are directly involved in the attachment mechanism. While the infundibulum is the portion of the sucker that comes into contact with the substrate, the acetabular protuberance should come into contact with the upper part of the side wall of the orifice to contribute to the production of efficient attachment over long time periods [14]. These findings and considerations agree well with the previously proposed idea [33] that a viscoelastic material serves as a type of damper, in which the relaxation response aids in the surface replication and optimization of a real contact between biological tissue and another biological or artificial substrate. Such a consideration could also explain why most pressure-sensitive adhesives have viscoelastic properties [40–46]. With this in mind, we may consider the RTC to be a sort of compliancy indicator: the shorter the RTC is, the greater the compliance of a biological tissue to a substrate. The time constant **τ**_2_ is lower in the infundibulum than in the acetabular protuberance. In contrast to the acetabular protuberance, which is an internal sucker structure, the infundibulum must be as compliant to the substrates as possible.

To the best of our knowledge, the mechanical data obtained from our investigation of octopus-sucker tissues are the first ones to be available in the literature and will be crucially important in allowing the mimicry of strategic morphological features of natural suckers in bioinspired artificial suction cups. We conclude that the properties of the common elastomers that are generally used in industrial suckers are quite dissimilar to the properties of biological suckers. In our opinion, existing materials that could better mimic the octopus-sucker tissues are soft hydrogels [47–50] and soft polyurethanes [51], as both of these types of material are in fact characterized by viscoelastic properties and have elasticity moduli of 0.1–10 and 100 kPa, respectively. To create an innovative attachment device inspired by octopus suckers, two types of material are needed: one softer and viscous to mimic the infundibular portion and one more elastic and less soft to mimic the acetabular protuberance. The first is important to replicate the amazing capability of the octopus to be compliant with any non-porous surfaces under wet conditions. This material should also be able to replicate the strategic structure (roughness and grooves) of the infundibular surface to maximize the attachment area. The other material should be exploited to design an efficient on–off attachment system (for example, one that is able to store elastic energy) to minimize the energy consumption for attachment over a long period of time.

## References

[1] Mazzolai B Margheri L Cianchetti M Dario P Laschi C 2012 Soft-robotic arm inspired by the octopus. II. From artificial requirements to innovative technological solutions. Bioinspir. Biomim. 7, 025005 (10.1088/1748-3182/7/2/025005) 22617166

[2] Laschi C Mazzolai B Mattoli V Cianchetti M Dario P 2009 Design of a biomimetic robotic octopus arm. Bioinspir. Biomim. 4, 015006 (10.1088/1748-3182/4/1/015006) 19258690

[3] Walker ID 2005 Continuum robot arms inspired by cephalopods. Proceedings of the SPIE 5804, 303–314. (10.1117/12.606201)

[4] Martinez RV Branch JL Fish CR Jin L Shepherd RF Nunes RMD Suo Z Whitesides GM 2013 Robotic tentacles with three-dimensional mobility based on flexible elastomers. Adv. Mater. 25, 205–212. (10.1002/adma.201203002) 22961655

[5] Cianchetti M Arienti A Follador M Mazzolai B Dario P Laschi C 2011 Design concept and validation of a robotic arm inspired by the octopus. Mater. Sci. Eng. C 31, 1230–1239. (10.1016/j.msec.2010.12.004)

[6] Laschi C Cianchetti M Mazzolai B Margheri L Follador M Dario P 2012 A soft robot arm inspired by the octopus. Adv. Robot. 26, 709–727. (10.1163/156855312X626343)

[7] Calisti M Arienti A Giannaccini ME Follador M Giorelli M Cianchetti M Mazzolai B Laschi C Dario P 2010 Study and fabrication of bioinspired octopus arm mockups tested on a multipurpose platform. In Biomedical Robotics and Biomechatronics (BioRob), 2010 3rd IEEE RAS and EMBS Int. Conf. 26–29 September, Tokyo, pp. 461–466. New York, NY: IEEE.

[8] Grasso FW Setlur P 2007 Inspiration, simulation and design for smart robot manipulators from the sucker actuation mechanism of cephalopods. Bioinspir. Biomim. 2, S170 (10.1088/1748-3182/2/4/S06) 18037726

[9] Hu B-s Wang L-w Fu Z Zhao Y-z 2009 Bio-inspired miniature suction cups actuated by shape memory alloy. Int. J. Adv. Robotic Sy. 6, 151–160.

[10] Hou J Wright E Bonser RHC Jeronimidis G 2012 Development of biomimetic squid-inspired suckers. J. Bionic Eng. 9, 484–493. (10.1016/S1672-6529(11)60144-3)

[11] Tramacere F Beccai L Mazzolai B 2013 What can we learn from the octopus? In Biological and biomimetic adhesives (eds Santos R Aldred N Gorb S Flammang P), pp. 89–102. Cambridge, UK: The Royal Society of Chemistry.

[12] Kier WM Smith AM 1990 The morphology and mechanics of octopus suckers. Biol. Bull. 178, 126–136. (10.2307/1541971) 29314931

[13] Kier WM Smith AM 2002 The structure and adhesive mechanism of octopus suckers. Integr. Comp. Biol. 42, 1146–1153. (10.1093/icb/42.6.1146) 21680399

[14] Tramacere F Beccai L Kuba M Gozzi A Bifone A Mazzolai B 2013 The morphology and adhesion mechanism of *Octopus vulgaris* suckers. PLoS ONE 8, e65074 (10.1371/journal.pone.0065074) 23750233 PMC3672162

[15] Smith AM 1991 Negative pressure generated by octopus suckers: a study of the tensile strength of water in nature. J. Exp. Biol. 157, 257–271.

[16] Smith AM 1996 Cephalopod sucker design and the physical limits to negative pressure. J. Exp. Biol. 199, 949–958. 9318745 10.1242/jeb.199.4.949

[17] Grasso FW 2008 Octopus sucker-arm coordination in grasping and manipulation. Am. Malacol. Bull. 24, 13–23. (10.4003/0740-2783-24.1.13)

[18] Scherge M Gorb SN 2001 Biological micro- and nanotribology: Nature‘s solutions. Berlin, Germany: Springer.

[19] Gorb SN 2007 Smooth attachment devices in insects: functional morphology and biomechanics. In Advances in insect physiology (eds Casas J Simpson SJ), pp. 81–115. Amsterdam, The Netherlands: Elsevier.

[20] Hertz H 1881 Über den Kontakt elastischer Körper. J. Reine Angew. Math. 92, 156–171.

[21] Wainwright SA Biggs WD Currey JD Gosline JM 1976 Mechanical design in organisms. Princeton, NJ: Princeton University Press.

[22] Vincent J 1990 Structural biomaterials. Princeton, NJ: Princeton University Press.

[23] Kier WM Smith KK 1985 Tongues, tentacles and trunks: the biomechanics of movement in muscular-hydrostats. Zool. J. Linn. Soc. 83, 307–324. (10.1111/j.1096-3642.1985.tb01178.x)

[24] Vogel S 2003 Comparative biomechanics: life‘s physical world. Oxford, UK: Princeton University Press.

[25] van Dommelen JAW van der Sande TPJ Hrapko M Peters GWM 2010 Mechanical properties of brain tissue by indentation: interregional variation. J. Mech. Behav. Biomed. Mater. 3, 158–166. (10.1016/j.jmbbm.2009.09.001) 20129415

[26] Samani A Zubovits J Plewes D 2007 Elastic moduli of normal and pathological human breast tissues: an inversion-technique-based investigation of 169 samples. Phys. Med. Biol. 52, 1565–1576. (10.1088/0031-9155/52/6/002) 17327649

[27] van Kuilenburg J Masen MA van der Heide E 2012 Contact modelling of human skin: what value to use for the modulus of elasticity? Proc. Inst. Mech. Eng. J. 227, 349–361. (10.1177/1350650112463307)

[28] Lang RM Cholley BP Korcarz C Marcus RH Shroff SG 1994 Measurement of regional elastic properties of the human aorta. A new application of transesophageal echocardiography with automated border detection and calibrated subclavian pulse tracings. Circulation 90, 1875–1882. (10.1161/01.CIR.90.4.1875) 7923675

[29] Thubrikar M Piepgrass WC Bosher LP Nolan SP 1980 The elastic modulus of canine aortic valve leaflets *in vivo* and *in vitro*. Circ. Res. 47, 792–800. (10.1161/01.RES.47.5.792) 7418136

[30] Millard L Espino DM Shepherd DET Hukins DWL Buchan KG 2011 Mechanical properties of chordae tendineae of the mitral heart valve: Young's modulus, structural stiffness, and effects of aging. J. Mech. Med. Biol. 11, 221–230. (10.1142/S0219519411003971)

[31] Barnes WJP Goodwyn PJP Nokhbatolfoghahai M Gorb SN 2011 Elastic modulus of tree frog adhesive toe pads. J. Comp. Physiol. A 197, 969–978. (10.1007/s00359-011-0658-1) PMC3176399 21667266

[32] Jiao Y Gorb S Scherge M 2000 Adhesion measured on the attachment pads of *Tettigonia viridissima* (Orthoptera, insecta). J. Exp. Biol. 203, 1887–1895. 10821745 10.1242/jeb.203.12.1887

[33] Gorb S Jiao Y Scherge M 2000 Ultrastructural architecture and mechanical properties of attachment pads in *Tettigonia viridissima* (Orthoptera Tettigoniidae). J. Comp. Physiol. A 186, 821–831. (10.1007/s003590000135) 11085636

[34] Perez Goodwyn P Peressadko A Schwarz H Kastner V Gorb S 2006 Material structure, stiffness, and adhesion: why attachment pads of the grasshopper (*Tettigonia viridissima*) adhere more strongly than those of the locust (*Locusta migratoria*) (Insecta: Orthoptera). J. Comp. Physiol. A 192, 1233–1243. (10.1007/s00359-006-0156-z) 16868765

[35] Santos R Gorb S Jamar V Flammang P 2005 Adhesion of echinoderm tube feet to rough surfaces. J. Exp. Biol. 208, 2555–2567. (10.1242/jeb.01683) 15961742

[36] Sanjeevi R 1982 A viscoelastic model for the mechanical properties of biological materials. J. Biomech. 15, 107–109. (10.1016/0021-9290(82)90042-2) 7076685

[37] Mak AF 1986 Unconfined compression of hydrated viscoelastic tissues: a biphasic poroviscoelastic analysis. Biorheology 23, 371–383. 3779062 10.3233/bir-1986-23406

[38] Flaud P Quemada D 1988 A structural viscoelastic model of soft tissues. Biorheology 25, 95–105. 3196840 10.3233/bir-1988-251-216

[39] Neville AC 1993 Biology of fibrous composites. Cambridge, UK: Cambridge University Press.

[40] Chang EP 1997 Viscoelastic properties of pressure-sensitive adhesives. J. Adhes. 60, 233–248. (10.1080/00218469708014421)

[41] Feldstein MM Bermesheva EV Jean YC Misra GP Siegel RA 2011 Free volume, adhesion, and viscoelastic properties of model nanostructured pressure-sensitive adhesive based on stoichiometric complex of poly(N-vinyl pyrrolidone) and poly(ethylene glycol) of disparate chain lengths. J. Appl. Polym. Sci. 119, 2408–2421. (10.1002/app.32917)

[42] Creton C Lakrout H 2000 Micromechanics of flat-probe adhesion tests of soft viscoelastic polymer films. J. Polym. Sci. B Polym. Phys. 38, 965–979. (10.1002/(SICI)1099-0488(20000401)38:7%3C965::AID-POLB7%3E3.0.CO;2-8)

[43] Miquelard-Garnier G Creton C Hourdet D 2007 Synthesis and viscoelastic properties of hydrophobically modified hydrogels. Macromol. Symp. 256, 189–194. (10.1002/masy.200751021)

[44] Carelli C Déplace F Boissonnet L Creton C 2007 Effect of a gradient in viscoelastic properties on the debonding mechanisms of soft adhesives. J. Adhes. 83, 491–505. (10.1080/00218460701377701)

[45] Glassmaker N Hui C Yamaguchi T Creton C 2008 Detachment of stretched viscoelastic fibrils. Eur. Phys. J. E 25, 253–266. (10.1140/epje/i2007-10287-y) 18398567

[46] Martina D Creton C Damman P Jeusette M Lindner A 2012 Adhesion of soft viscoelastic adhesives on periodic rough surfaces. Soft Matter 8, 5350–5357. (10.1039/c2sm07059f)

[47] Ahearne M Yang Y Liu KK 2008 Mechanical characterisation of hydrogels for tissue engineering applications. In Topics in tissue Engineering (eds Ashammakhi N Reis R Chiellini F), Ch. 12, Vol. 4, Expertissues series, available at: http://www.oulu.fi/spareparts/ebook_topics_in_t_e_vol4/published_chapters.html.

[48] Ahearne M Yang Y El Haj AJ Then KY Liu K-K 2005 Characterizing the viscoelastic properties of thin hydrogel-based constructs for tissue engineering applications. J. R. Soc. Interface 2, 455–463. (10.1098/rsif.2005.0065) 16849205 PMC1618501

[49] Banerjee A Arha M Choudhary S Ashton RS Bhatia SR Schaffer DV Kane RS 2009 The influence of hydrogel modulus on the proliferation and differentiation of encapsulated neural stem cells. Biomaterials 30, 4695–4699. (10.1016/j.biomaterials.2009.05.050) 19539367 PMC2743317

[50] Nemir S Hayenga HN West JL 2010 PEGDA hydrogels with patterned elasticity: novel tools for the study of cell response to substrate rigidity. Biotechnol. Bioeng. 15, 636–644. (10.1002/bit.22574) 19816965

[51] Santos D Spenko M Parness A Kim S Cutkosky M 2007 Directional adhesion for climbing: theoretical and practical considerations. J. Adhes. Sci. Technol. 21, 1317–1341. (10.1163/156856107782328399)

